# Supplementary material for: Incidences of problematic cell lines are lower in papers that use RRIDs to identify cell lines
Source: eLife. 2019 Jan 29;8:e41676. doi: 10.7554/eLife.41676 (PMC6351100; doi:10.7554/eLife.41676)
Supplement: Transparent reporting form [file elife-41676-transrepform.docx]

***eLife’s* transparent reporting form**

We encourage authors to provide detailed information *within their submission* to facilitate the interpretation and replication of experiments. Authors can upload supporting documentation to indicate the use of appropriate reporting guidelines for health-related research (see [EQUATOR Network](http://www.equator-network.org/%20)), life science research (see the [BioSharing Information Resource](https://biosharing.org/)), or the [ARRIVE guidelines](http://www.plosbiology.org/article/info:doi/10.1371/journal.pbio.1000412) for reporting work involving animal research. Where applicable, authors should refer to any relevant reporting standards documents in this form.

If you have any questions, please consult our Journal Policies and/or contact us: [editorial@elifesciences.org](mailto:editorial@elifesciences.org).

**Sample-size estimation**

- You should state whether an appropriate sample size was computed when the study was being designed
- You should state the statistical method of sample size computation and any required assumptions
- If no explicit power analysis was used, you should describe how you decided what sample (replicate) size (number) to use

Please outline where this information can be found within the submission (e.g., sections or figure legends), or explain why this information doesn’t apply to your submission:

Our samples were selected based on the complete population of articles that was available in PMC. Our tools tested every accessible open access paper, ~2 million, and every paper in which authors published an RRID for a cell line, ~1,500. We consulted with the UCSD statistician on call, just to make sure that we were using appropriate statistical tests.

**Replicates**

- You should report how often each experiment was performed
- You should include a definition of biological versus technical replication
- The data obtained should be provided and sufficient information should be provided to indicate the number of independent biological and/or technical replicates
- If you encountered any outliers, you should describe how these were handled
- Criteria for exclusion/inclusion of data should be clearly stated
- High-throughput sequence data should be uploaded before submission, with a private link for reviewers provided (these are available from both GEO and ArrayExpress)

Please outline where this information can be found within the submission (e.g., sections or figure legends), or explain why this information doesn’t apply to your submission:

Our initial training data for the cell line recognition algorithm was created by Mr. Menke, and reviewed by Dr. Bandrowski. This training data set was verified by Dr. Bairoch. The algorithm was trained 10 times and we report the average F1-score:

“The human-curated data were split into 90%/10% datasets for training and testing. The data was split, and the SciScore was trained ten10 times, the average values for precision, recall and the harmonic mean (F1-score) are reported.”

Once we completed the run on the ~2 million open access papers, we sent the initial report of cell line names to our co-authors, Drs. Capes-Davis and Bairoch, who re-analyzed the data. We report in the manuscript that this process led to some important additions to the Cellosaurus database, and as a result the current version of Cellosaurus now includes these additions.

**Statistical reporting**

- Statistical analysis methods should be described and justified
- Raw data should be presented in figures whenever informative to do so (typically when N per group is less than 10)
- For each experiment, you should identify the statistical tests used, exact values of N, definitions of center, methods of multiple test correction, and dispersion and precision measures (e.g., mean, median, SD, SEM, confidence intervals; and, for the major substantive results, a measure of effect size (e.g., Pearson's r, Cohen's d)
- Report exact p-values wherever possible alongside the summary statistics and 95% confidence intervals. These should be reported for all key questions and not only when the p-value is less than 0.05.

Please outline where this information can be found within the submission (e.g., sections or figure legends), or explain why this information doesn’t apply to your submission:

We were initially concerned because we proposed to use the z statistic, thus we conferred with the statistician on call at UCSD math department. He confirmed that the t and z tests converge far earlier than 1000 samples so these two tests are essentially identical when comparing >1000 samples. We therefore used the z-score to report difference in means.

We used standard measures for classification performance: precision P, recall R and harmonic mean of precision and recall F1, the formula is included at the end of the methods section in the manuscript.

(For large datasets, or papers with a very large number of statistical tests, you may upload a single table file with tests, Ns, etc., with reference to sections in the manuscript.)

**Group allocation**

- Indicate how samples were allocated into experimental groups (in the case of clinical studies, please specify allocation to treatment method); if randomization was used, please also state if restricted randomization was applied
- Indicate if masking was used during group allocation, data collection and/or data analysis

Please outline where this information can be found within the submission (e.g., sections or figure legends), or explain why this information doesn’t apply to your submission:

Our sample was allocated based on the total available data. We did not throw away any paper in PubMed Central that was available for text mining at the time that we downloaded the data. The sample for RRID papers included every paper that was published in any journal where the author indicated that the cell line using the RRID syntax. We removed articles that were not associated with the PubMed Identifier, PMID. The reasons that a paper would not have an PMID fall into the following: the paper may have been curated before it was submitted to PubMed (early online version); the paper may be published in a journal that is not indexed by PubMed, or the paper may be a copy of another paper that is in a strange format therefore SciBot did not find the PubMed Id, and curators may have already found the paper in a different format. It is much more likely that the paper without a PubMed ID is a duplicate or is published in a predatory journal. We routinely exclude such papers from all of SciCrunch pipelines.

**Additional data files (“source data”)**

- We encourage you to upload relevant additional data files, such as numerical data that are represented as a graph in a figure, or as a summary table
- Where provided, these should be in the most useful format, and they can be uploaded as “Source data” files linked to a main figure or table
- Include model definition files including the full list of parameters used
- Include code used for data analysis (e.g., R, MatLab)
- Avoid stating that data files are “available upon request”

Please indicate the figures or tables for which source data files have been provided:

The data underlying all of the figures and references in the text are available as an excel file called Figure.Data and these are broken down by worksheets that correspond to each relevant figure. Data for each RRID paper and each cell line is available from Scicrunch.org/resolver/[RRID], e.g., <https://scicrunch.org/resolver/RRID:CVCL_0058> or <https://scicrunch.org/resolver/RRID:CVCL_0058.xml> for developers, and from the CrossRef Event database in the W3C open annotation standard under a CC-0 license. SciScore was recently released to the public at sciscore.com, and in the next few months will be available to all authors as a free tool for manuscript verification.
